# Effect of Magnesium-Modified Titanium Implants on Osseointegration: A Systematic Review and Meta-Analysis of Preclinical Studies

**DOI:** 10.3390/jcm15051987

**Published:** 2026-03-05

**Authors:** Ali Alenezi, Dhafer Alasmari

**Affiliations:** 1 Department of Prosthetic Dental Sciences, College of Dentistry, Qassim University, Buraydah 51452, Saudi Arabia; 2 Department of Periodontology and Implant Dentistry, College of Dentistry, Qassim University, Buraydah 51452, Saudi Arabia

**Keywords:** magnesium coating, titanium dental implants, osseointegration, surface modification, systematic review, meta-analysis

## Abstract

**Objectives**: This study systematically evaluated and quantitatively synthesized preclinical evidence on the effects of magnesium (Mg) incorporation into or coating of titanium dental implants on osseointegration and peri-implant bone formation. **Methods**: Electronic searches of PubMed, Scopus, and Web of Science were performed up to May 2025 to identify animal studies evaluating Mg-modified titanium implants. Eligible studies compared Mg-incorporated or Mg-coated implants with non-modified titanium controls and reported quantitative histomorphometric outcomes. Primary outcomes included the values of bone-to-implant contact (BIC) and bone area (BA) around implants. Study quality was assessed using the ARRIVE 2.0 guidelines. Meta-analyses were performed using weighted mean differences with 95% confidence intervals under fixed- or random-effects models based on heterogeneity. **Results**: Eleven preclinical animal studies conducted in rabbit and rat models were included. Mg was incorporated using various surface-modification techniques, including ion implantation, Mg-substituted hydroxyapatite coatings, mesoporous titania layers, and nanotubular structures. Overall, the studies’ quality was high, with most studies rated as excellent and with a low-to-moderate risk of bias. Furthermore, the meta-analysis revealed a significant increase in BIC for Mg-modified implants compared with uncoated implants (Z = 4.38, *p* < 0.001), implying improved osseointegration. Meanwhile, pooled BA values showed no significant differences between the groups (Z = 0.93, *p* = 0.35). **Conclusions**: Mg coating onto or incorporation into titanium implant surfaces can improve BIC in preclinical models, indicating improved osseointegration in the early stages.

## 1. Introduction

Dental implants are the favored treatment option for replacing missing teeth in many cases, with success rates exceeding 90% in one long-term evaluation [1,2]. The cornerstone of implant success is the establishment of osseointegration, defined as a direct, load-bearing interface between bone and an implant surface without intervening fibrous tissue [3,4]. However, systemic and local risk factors, such as osteoporosis, diabetes mellitus, radiation therapy, smoking, and peri-implant infections, may negatively affect bone healing and increase the risk of biological complications or early implant failure [5]. Therefore, advances in implant surface engineering aimed at enhancing biological responses at the bone–implant interface have remained a major research priority.

Various surface modification approaches, such as micro-roughening, sandblasting, acid-etching, anodization, and laser structuring, have been developed to improve cellular adhesion, accelerate bone apposition, and enhance early implant stability [6,7]. Moreover, bioactive implant coatings, such as hydroxyapatite (HA), have been investigated extensively due to their chemical nature being similar to natural bone minerals [4]. However, some reports raised concerns related to their long-term reliability and limited clinical success [8]. Recently, there have been more attempts to incorporate ions or elements using new strategies to enhance osteogenic and immune responses at the molecular level.

Magnesium (Mg) has recently emerged as one of the most promising elements for dental implant surface functionalization [9,10]. As the fourth most abundant positively charged ion in the human body, Mg plays an essential role in bone metabolism, cellular homeostasis, osteoblast differentiation, and mineral deposition [11]. Strong evidence suggests that Mg ions can stimulate crucial pro-osteogenic pathways—including Wnt/β-catenin and VEGF/HIF-1α—thereby enhancing angiogenesis and improving bone tissue formation around implants [12]. Additionally, Mg exhibits immunomodulatory properties by reducing inflammatory cytokine activity and favoring M2 macrophage polarization, ultimately supporting a regenerative peri-implant environment [13].

Mg has been extensively investigated as an implant coating due to it sustained biodegradability, which permits gradual ion release directly at the bone–implant interface [14,15]. The localized release of Mg ions has been shown to enhance osteoblast proliferation, control osteoclast differentiation, and limit bacterial adhesion on implant surfaces [16]. These activates can facilitate osseointegration and improve resistance to implant-associated infections [16,17]. These effects can be crucial since peri-implant mucositis and peri-implantitis are major causes of implant loss in the long term [18].

Several preclinical reports revealed the favorable effects of Mg incorporation or coatings on implant osseointegration. For instance, Tao et al. reported that Mg-, Zn-, and Sr-incorporated HA coatings can significantly enhance bone formation in osteopenic rats [19]. Yang et al. reported that Mg-incorporated Ti nanotube implants demonstrated better integration with bone with lower risks of infection-induced osteolysis [20]. Furthermore, Shen et al. found higher bone regeneration and antibacterial activities when coating implant with a Mg/zinc framework [21]. Some reports suggested that titanium implants modified with Mg with different microstructures have a positive influence on implant integration with bone especially in compromised bone conditions [9]. This was confirmed by some in vivo studies that found greater bone formation and stronger biomechanical anchorage in osteoporotic animal models [9,17]. In their in vivo investigation, Okuzu et al. reported improved early stability for implants with Mg- and Sr-releasing surfaces in a rabbit implant model [22]. Another animal experiment investigated titanium (Ti) implants coated with Mg ions and reported enhanced peri-implant bone quality compared with control implants without a coating in osteoporotic bone [23].

However, in addition to these favorable findings, other studies found conflicting results. For instance, Galli et al. found that Mg-releasing mesoporous coatings did not significantly influence early bone maturation in rabbits [24]. Variations in coating techniques, including micro-arc oxidation, ion implantation, sol–gel processing, and nanoscale deposition, may result in differing Mg release kinetics, degradation behavior, and biological responses [25,26]. Accordingly, the available research evidence remains fragmented due to heterogeneous study designs, animal models, coating formulations, and outcome evaluation methods.

Given the increasing adoption of Mg-incorporated implant technologies and the need to apply these findings in human patients, a systematic and quantitative appraisal of the existing evidence is warranted. Therefore, this systematic review and meta-analysis aims to comprehensively assess the effects of Mg-coated dental implants on osseointegration while exploring potential sources of heterogeneity across preclinical studies.

## 2. Results

**Figure 1:**
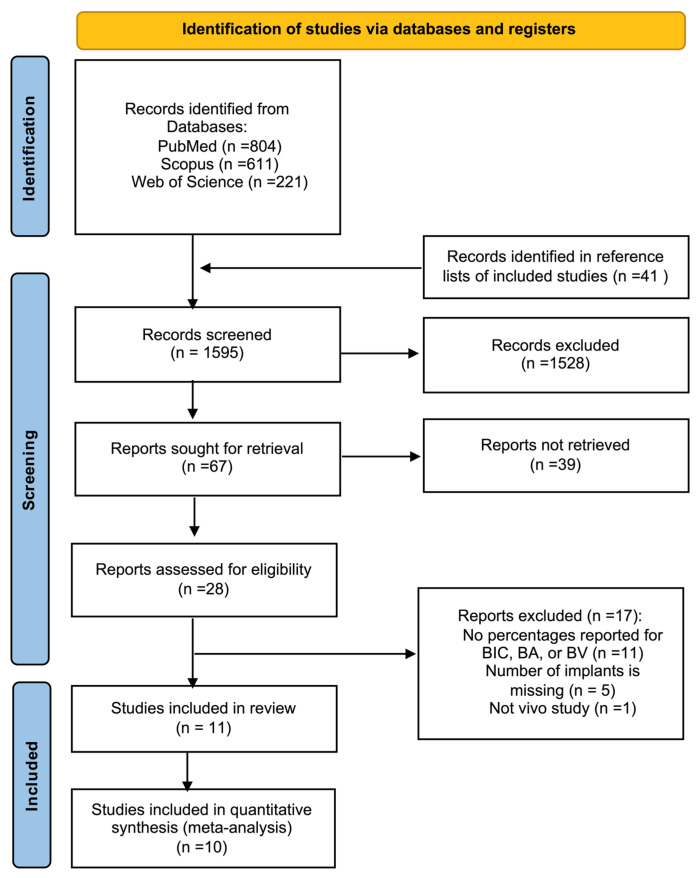
PRISMA flow diagram showing the search process for this systematic review.

Eleven preclinical animal studies that evaluated Mg-incorporated or Mg-coated Ti implants were included in this review (Figure 1) [19,20,21,22,24,27,28,29,30,31,32]. The implants were evaluated in rabbit or rat models, primarily in tibial and femoral bone sites, with healing periods of 2–24 weeks. Multiple surface-modification strategies are represented, including Mg-ion implantation, Mg-substituted HA coatings, Mg-loaded mesoporous titania layers, and Mg-incorporated nanotubular structures (Table 1).

### 2.1. Quality Assessment

**Figure 2:**
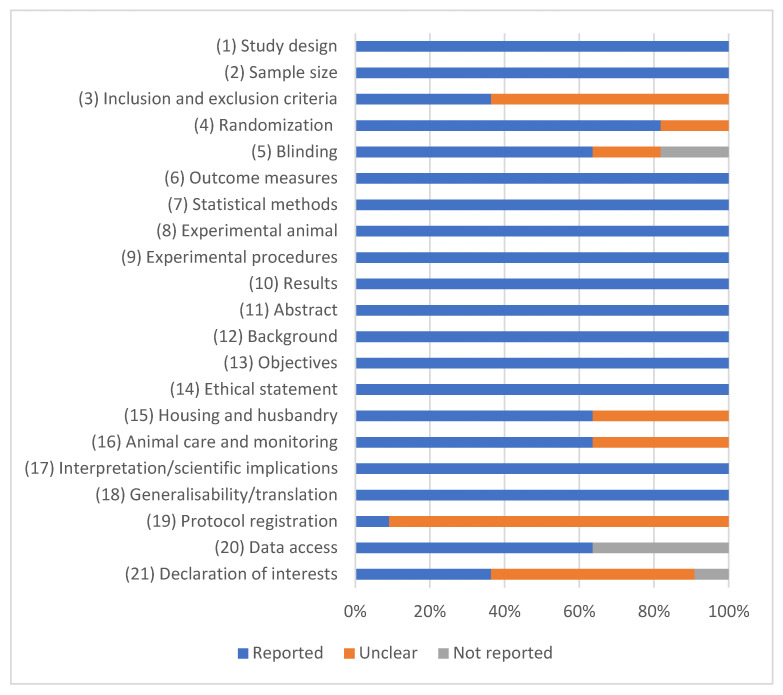
Quality assessments of the studies based on ARRIVE 2.0 guidelines. Obtained results are expressed as percentages.

An ARRIVE 2.0 evaluation revealed an overall high study quality, with eight studies rated as excellent and three studies rated as average (Figure 2; Table 2). No studies were excluded due to quality concerns.

### 2.2. Risk-of-Bias Assessment

**Figure 3:**
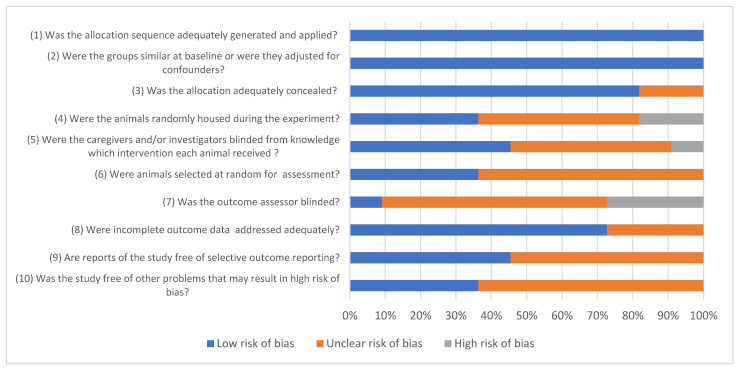
Risk-of-bias distribution based on SYRCLE assessment. Obtained results are presented as percentages.

The risk-of-bias assessment using the SYRCLE tool revealed an overall low-to-moderate risk of bias across the included preclinical studies (Figure 3). Most of the studies demonstrated a low risk of bias for domains related to baseline characteristics, outcome assessment, and selective outcome reporting. Outcome data were generally complete, and histomorphometric analyses, such as BIC and BA fraction, were clearly defined and consistently reported.

### 2.3. Meta-Analysis of BIC

**Figure 4:**
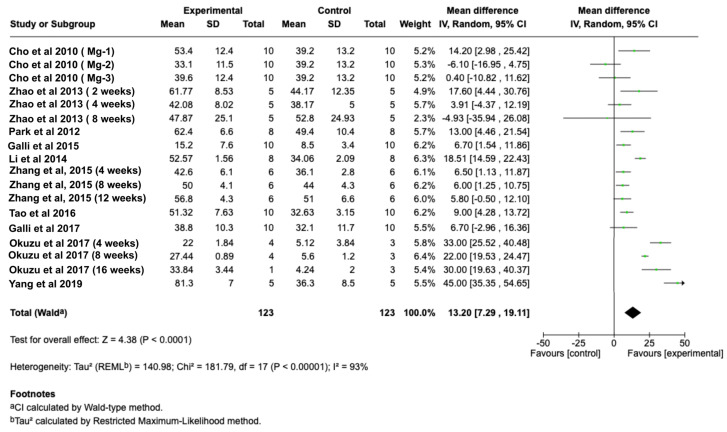
Forest plot showing the BIC values on Ti implants with Mg coatings (test) or without coatings (control). The pooled analysis showed a statistically significant overall effect for Mg-coated implants (Z = 4.38.01, *p* < 0.001) [19,20,22,24,27,28,29,30,31,32].

Quantitative synthesis demonstrated a significant increase in BIC for Mg-incorporated implants when compared with non-Mg controls. The pooled analysis showed a favorable effect of Mg-modified surfaces (Z = 4.38, *p* < 0.001) (Figure 4). Improvements in BIC were observed across the included studies; however, no formal subgroup analyses were conducted to assess the influence of specific coating strategies.

### 2.4. Meta-Analysis of BA

**Figure 5:**
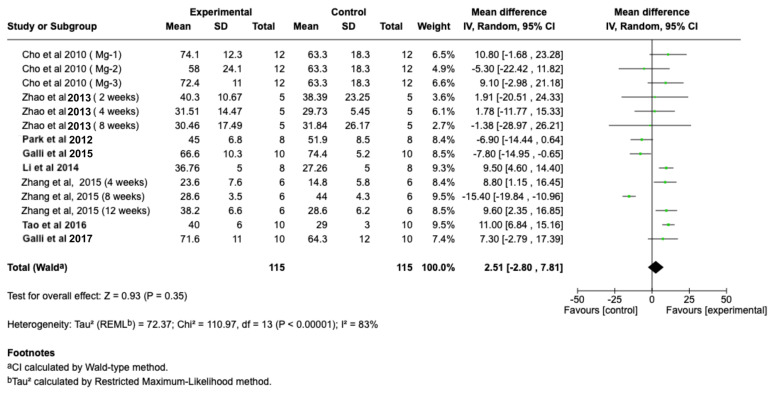
Forest plot showing BA values from the included studies on implants with Mg coatings (test) or without coatings (control). The pooled analysis revealed no significant difference between Mg-coated and control implants (Z = 0.93, *p* = 0.35) [19,24,27,28,29,30,31,32].

Pooled BA (%) outcomes did not show a significant difference within the experimental and control groups (Z = 0.93, *p* = 0.35) (Figure 5). However, a trend toward increased peri-implant bone formation was noted, particularly in studies with extended healing durations (≥8 weeks).

## 3. Discussion

This review demonstrates that Mg incorporation into Ti implant surfaces significantly enhances BIC, indicating improved early osseointegration compared with unmodified controls [19,20,21,22,24,27,28,29,30,31,32]. These findings are consistent with preclinical literature showing that Mg supports osteoblast function and mineralization by modulating integrin signaling, RUNX2 expression, and alkaline phosphatase activity [33,34]. Improved BIC may reflect Mg’s capacity to accelerate contact osteogenesis during early healing.

Although BA did not show a significant pooled improvement, this may be due to BIC and BA reflecting different biological phenomena: BIC captures the quality of direct bone contact at an implant interface, whereas BA represents regional bone volume that may require longer remodeling periods to change significantly [35]. The released Mg concentrates near implant surfaces, creating localized effects that significantly improve bone–implant contact without immediately changing the broader bone architecture [22]. Indeed, the studies using controlled-release systems, such as Mg-loaded mesoporous titania and nanotubes, reported enhanced bone volume, indicating more mature bone formation with optimized ion delivery [20,36].

Mg may also support osseointegration via immunomodulatory mechanisms by promoting M2 macrophage polarization and angiogenesis via HIF-1α and VEGF pathways [37,38]. Although few of the included studies assessed immune or vascular responses directly, the consistent increase in BIC supports the presence of a favorable early peri-implant environment [20,21].

Furthermore, a main cause of heterogeneity within the included studies is the variation in Mg coating methods. The evaluated studies used diverse techniques, such as ion implantation, micro-arc oxidation, mesoporous titania carriers, and nanotubular structures [39,40]. These methods produce coatings that differ considerably in coating features, degradation behavior, and surface topography. For instance, ion implantation or incorporation into the surface can produces thin layers with a controlled Mg concentration but reduced release duration [31]. Meanwhile, loading Mg into surfaces with mesoporous or nanotubular features may allow for sustained ion release, which may extend its biological activity [20]. It has been suggested that hydroxyapatite-based systems exhibit osteoconductive properties, though concerns were raised regarding degradation behavior and coating stability of HA in the long term. Since subgroup analyses were not performed in this review, definitive conclusions regarding the advantage of one method over another cannot be drawn. Nevertheless, the inconsistency in biological effects likely reflects differences in release behavior and surface stability between the implants produced using different modification techniques.

Another important consideration for Mg’s effects is their dose-dependence. Moderate Mg ion release is believed to promote osteogenesis and angiogenesis. However, high concentrations of Mg may enhance corrosion and be associated with cytotoxicity [41]. No comparisons were mentioned in the included studies regarding the optimal release behavior for Mg and the ideal therapeutic amount for Mg incorporation and release remains unclear.

From a clinical prospective, Mg-coated implants could provide significant advantages, especially in compromised bone conditions, such as osteoporosis, where improved osseointegration has been confirmed in preclinical models [19,31]. These results indicate that this is a promising surface modification method. However, concerns remain regarding coating stability and loading protocols, which require further investigation in clinical trials with functionally loaded jawbone models. In this review, the included studies were performed mainly on small animal models and without functional loading, and human clinical data remain absent. These promising preclinical findings require validation using large-animal studies and clinical evaluation before general implementation in dental practice.

Limitations of this study include methodological heterogeneity across studies and inadequate reporting of biomechanical outcomes. Furthermore, the ideal Mg concentration and release profiles remain uncertain, since high Mg concentrations may lead to corrosion or cytotoxicity [15,42]. Future studies should aim to evaluate standardize concentration levels and evaluate dose–response effects under controlled experimental conditions.

## 4. Materials and Methods

### 4.1. PICO Framework

This systematic review was according to the guidelines of the Preferred Reporting Items for Systematic Reviews and Meta-Analyses (PRISMA) (Supplementary Table S1) [43]. Additionally, it was recorded in the International Platform of Registered Systematic Review and Meta-Analysis Protocols (INPLASY) (registration number: INPLASY202610101). The PICO framework was established at beginning of the search process as follows:

*Population (P)*: animal models;
*Intervention (I)*: Ti implants coated or incorporated with Mg;
*Control (C)*: Ti implants without Mg incorporation or coating;
*Outcome (O)*: bone formation around implants.

### 4.2. Search Strategies

An electronic search was conducted in May 2025 in three main databases (PubMed, Scopus, and Web of Science) to identify animal studies published not before 2005. The search terms included four key concepts: bone formation, Ti implants, Mg coating, and animal experimentation. The search strategy was as follows: (“bone formation” OR “bone remodeling” OR “osseointegration”) AND (“titanium implants” OR “bone-implant interface”) AND (“magnesium” OR “Mg coating” OR “antimicrobial surfaces” OR “implant surface modification”) AND (“animal models” OR “in vivo”).

### 4.3. Inclusion and Exclusion Criteria

Studies were eligible if they investigated Mg-modified endosseous Ti implants in animal models. The Mg had to be applied locally, either as a coating or as a releasable agent from an implant surface, during or before implant insertion. Eligible studies reported quantitative bone outcomes, such as bone-to-implant contact (BIC), the percent bone volume (BV/TV), or bone area (BA). The uncoated (control) and Mg-modified (test) implants groups were required to have implants with similar surface characteristics except for the incorporation of Mg. Only articles published in English were included.

### 4.4. Study Selection

Titles and abstracts retrieved from the search results were screened against the eligibility criteria. Two independent reviewers evaluated the abstracts, and when relevance was uncertain, the full-text articles were assessed.

### 4.5. Data Extraction

A standardized form was used for data extraction to collect information related to study characteristics and publication date. The collected experimental information included animal species, number of subjects and implants, healing duration, and Mg-coating technique. Furthermore, the mean ± SD values for BIC and BA results for both the test and control groups were collected.

### 4.6. Quality Assessment

Study quality was determined using the ARRIVE 2.0 reporting guidelines for animal research [44]. These guidelines include 21 evaluation items. Every item was scored 2 points (reported), 1 point (unclear), or 0 points (not reported). Following that, a quality coefficient was calculated to categorize the studies as excellent (0.8–1), average (0.5–0.8), or poor (<0.5).

### 4.7. Risk-of-Bias Assessment

The SYRCLE risk-of-bias tool was used to assess potential biases and evaluate methodological reliability among the included experimental studies [45].

### 4.8. Data Analysis

The analysis examined two quantitative measurements: BA and BIC percentages. In the statistical calculations, weighted mean differences used the number of implants as the unit of analysis. Studies lacking adequate reporting of the primary outcomes were excluded. Heterogeneity was assessed using the I^2^ statistic. For the heterogeneity, a random-effects with inverse-variance weighting was used when significant heterogeneity was found; otherwise, a fixed-effects approach was applied. The effect size was reported as the mean difference with 95% confidence intervals. All statistical tests were conducted using Review Manager software (Version 5.3.3, The Nordic Cochrane Centre).

## 5. Conclusions

This systematic review and meta-analysis revealed that incorporating Mg or coating Ti implant surfaces with Mg may significantly improve BIC, indicating enhanced early tissue integration at an implant interface. Although the pooled BA data did not show a statistically significant difference, trends toward increased peri-implant bone formation were observed in several studies, particularly when Mg was delivered using controlled-release coating systems.

These findings collectively highlight the beneficial role of Mg in accelerating osseointegration and improving the quality of interfacial bone contact around Ti implants. Nevertheless, all the included studies were conducted in small-animal long-bone models, with limited evaluation of functional loading or long-term stability. Future research in jawbone environments and well-designed clinical trials is necessary to confirm the translatability of these promising preclinical outcomes into routine dental implant practice.

## Figures and Tables

**Table 1:** Overview of the included studies in this systematic review.

Authors	Coating Technique	Animal Model	Implant Site	Evaluation Method(s)	Healing Period	Control Group	Test Group(s)
Cho et al. 2010 [27]	Mg ion-implanted implants treated with resorbable blasting media (RBM)	New Zealand White rabbits	Tibia	BIC and BA	6 weeks	Ti implants treated with RBM	Implants incorporating Mg-1 (a), Mg-2 (b), Mg-3 (c)
Zhao et al. 2013 [28]	Magnesium-substituted nanohydroxyapatite coating on implant	New Zealand White rabbits	Femurs	BIC and BA	2, 4, and 8 weeks	Electrochemically deposited pure hydroxyapatite (EDHA) coatings on the surface of Ti implants	Electrochemically deposited magnesium-substituted hydroxyapatite (EDMHA) coatings on the surface of pure Ti implants
Park et al. 2012 [29]	Commercial microstructured Ti implants incorporating magnesium	New Zealand White rabbits	Femurs	BIC and BA	4 weeks	Ti implants produced by hydroxyapatite grit blasting (RBM implant)	Ti implants with Mg-incorporated nanoporous oxide layer (RBM/ Mg implant)
Galli et al. 2015 [24]	Mesoporous titania surfaces loaded with magnesium	New Zealand White rabbits	Tibia	BIC and BA	3 weeks	Ti implants coated with thin films of mesoporous TiO_2_	Mg-loaded mesoporous implants
Li et al. 2014 [30]	Magnesium-incorporated hydroxyapatite (HA) coating	Sprague Dawley rats	Femurs	BIC and BA	12 weeks	HA-coated implants	Mg-HA coated implants
Zhang et al. 2015 [31]	Hydroxyapatite (HA) coating with zinc (Zn), magnesium (Mg), or strontium (Sr)	Ovariectomized Sprague Dawley rats	Tibia	BIC and BA	4, 8, and 12 weeks	HA coating	Zn-HA, Mg-HA, and Sr-HA coatings
Tao et al. 2016 [19]	Zinc, magnesium, strontium-incorporated hydroxyapatite-coated Ti implants	Sprague Dawley rats	Femurs	BIC, BA, and BV/TV	12 weeks	HA coating	Zn-HA, Mg-HA coatings
Galli et al. 2017 [32]	Ti implants were coated with mesoporous titania layers and loaded with Mg	New Zealand White rabbits	Tibia	BIC and BA	6 weeks	Control implants	Mg-loaded implants
Okuzu et al. 2017 [22]	Alkali and heat treatment followed by Mg (and Sr) ion incorporation	Japanese White rabbits	Tibia	BIC	4, 8, 16, and 24 weeks	Cp-Ti implants	Mg-Ti implants
Yang et al. 2019 [20]	Magnesium-incorporated Ti nanotubes	Rats	Femurs	BIC	35 d	Nanotube-modified Ti implants (NT)	(Mg)-incorporated NT implants (NT-Mg)
Shen et al. 2019 [21]	Magnesium/zinc metal–organic framework on Ti implants	Rats	Femurs	BV/TV	4 weeks	Alkali–heat-treated Ti (AT) implants	AT-Mg/Zn3 implants

**Table 2:** Quality coefficients of the included studies.

Authors	Year	Animal Model	Quality Coefficient	Category
Cho et al. [27]	2010	New Zealand White rabbits	0.833	Excellent
Zhao et al. [28]	2013	New Zealand White rabbits	0.738	Average
Park et al. [29]	2012	New Zealand White rabbits	0.833	Excellent
Galli et al. [24]	2015	New Zealand White rabbits	0.880	Excellent
Li et al. [30]	2014	Sprague Dawley rats	0.952	Excellent
Zhang et al. [31]	2015	Sprague Dawley rats	0.952	Excellent
Tao et al. [19]	2016	Sprague Dawley rats	0.833	Excellent
Galli et al. [32]	2017	New Zealand White rabbits	0.904	Excellent
Okuzu et al. [22]	2017	Japanese White rabbits	0.761	Average
Yang et al. [20]	2019	Rats	0.976	Excellent
Shen et al. [21]	2019	Rats	0.928	Excellent

## Data Availability

Data collection and interpretation in this study are maintained by the authors and available upon request.

## Supplementary Materials

The following supporting information can be downloaded at: https://www.mdpi.com/article/10.3390/jcm15051987/s1, Table S1: PRISMA checklist.

## Abbreviations

The following abbreviations are used in this manuscript:

Mg	magnesium
Ti	titanium
BIC	bone-to-implant contact
BA	bone area
PRISMA	Preferred Reporting Items for Systematic Reviews and Meta-Analyses
